# Predicting mild cognitive impairment among Chinese older adults: a longitudinal study based on long short-term memory networks and machine learning

**DOI:** 10.3389/fnagi.2023.1283243

**Published:** 2023-10-23

**Authors:** Yucheng Huang, Zishuo Huang, Qingren Yang, Haojie Jin, Tingke Xu, Yating Fu, Yue Zhu, Xiangyang Zhang, Chun Chen

**Affiliations:** ^1^School of Public Health and Management, Wenzhou Medical University, Wenzhou, Zhejiang, China; ^2^School of Innovation and Entrepreneurship, Wenzhou Medical University, Wenzhou, Zhejiang, China; ^3^The First Affiliated Hospital of Wenzhou Medical University, Wenzhou, Zhejiang, China; ^4^Center for Healthy China Research, Wenzhou Medical University, Wenzhou, Zhejiang, China

**Keywords:** mild cognitive impairment, machine learning (ML), LSTM (long short-term memory networks), prediction model, China

## Abstract

**Background:**

Mild cognitive impairment (MCI) is a transitory yet reversible stage of dementia. Systematic, scientific and population-wide early screening system for MCI is lacking. This study aimed to construct prediction models using longitudinal data to identify potential MCI patients and explore its critical features among Chinese older adults.

**Methods:**

A total of 2,128 participants were selected from wave 5–8 of Chinese Longitudinal Healthy Longevity Study. Cognitive function was measured using the Chinese version of Mini-Mental State Examination. Long- short-term memory (LSTM) and three machine learning techniques, including 8 sociodemographic features and 12 health behavior and health status features, were used to predict individual risk of MCI in the next year. Performances of prediction models were evaluated through receiver operating curve and decision curve analysis. The importance of predictors in prediction models were explored using Shapley Additive explanation (SHAP) model.

**Results:**

The area under the curve values of three models were around 0.90 and decision curve analysis indicated that the net benefit of XGboost and Random Forest were approximate when threshold is lower than 0.8. SHAP models showed that age, education, respiratory disease, gastrointestinal ulcer and self-rated health are the five most important predictors of MCI.

**Conclusion:**

This screening method of MCI, combining LSTM and machine learning, successfully predicted the risk of MCI using longitudinal datasets, and enables health care providers to implement early intervention to delay the process from MCI to dementia, reducing the incidence and treatment cost of dementia ultimately.

## 1. Introduction

With an increasing older adult population worldwide, geriatric health concerns cannot be ignored. Aging results in declining physical and cognitive functions, leading to a high risk of disability and death (Klimova et al., 2017). Distinguishing between pathological and normal cognitive decline, generally referred to as dementia or cognitive impairment, remains challenging. As an inevitable human phenomenon, aging is a significant factor in deteriorating cognitive function. With a global increase in life expectancy, older adults have an increased likelihood of developing dementia and cognitive impairment. The World Health Organization (WHO) stated that >55 million older adults had a diagnosis of dementia in 2021, with >139 million older adults estimated to be diagnosed with dementia in 2050 worldwide. In 2019, the annual cost of dementia-related treatment exceeded US $1.3 trillion (World Health Organization, 2021). China has the greatest population of people with dementia, comprising 25% of the global population. Aggregate expenditure on dementia in China reached US $195 billion in 2019 (Jia et al., 2020b; Mattap et al., 2022).

With no reversal therapies available, prevention of dementia remains a priority. Mild cognitive impairment (MCI), a risk factor for dementia, is considered a transitional stage between normal cognitive function and dementia, where there is objective cognitive decline but with a capacity to live independently. However, approximately 10–20% of older adults aged ≥65 years with MCI are diagnosed with dementia after 1 year (Langa and Levine, 2014). Delaying the progression of MCI to dementia is currently the most effective approach, as diverse treatments for MCI have proven to be effective and less costly (Langa and Levine, 2014; Anderson, 2019; Huang et al., 2022), with early identification and intervention in high-risk groups shown to prevent dementia onset in 40% of such cases.

Currently, screening techniques and questionnaires for MCI are limited. On account of the fact that neurodegenerative disease starts to develop many years before the symptoms are observed, while applying MCI screening to the population with normal cognitive function, imaging examinations, and fluid biomarkers can detect the neurodegenerative and pathological changes most accurately. Imaging techniques, such as magnetic resonance imaging (MRI), positron emission computed tomography (PET), and single photon emission computed tomography (SPECT), are capable of showing the tiny changes in brain structure, blood flow, metabolism, and neurotransmitters in patients with MCI. Nevertheless, due to the rarity and inaccessibility of these techniques for the general public, they cannot be used as a common screening tool for MCI (Dunne et al., 2021), with limited coverage in terms of MCI questionnaires [Mini-Mental State Examination (MMSE) and the Montreal Cognitive Assessment (MoCA)] that generally require a significant investment in manpower and their training. Therefore, an effective, systematic, and convenient MCI screening method to identify high-risk older adults in the general population is urgently needed. Effective screening could be conducive to targeted interventions for those at high risk of MCI. One study reported significant changes through implementing appropriate early intervention for potential patients in England, namely, an 8.5% decrease in the incidence of dementia and a reduction in dementia-related expenditure of approximately $180 million (Mukadam et al., 2020). Owing to the irreversible nature of dementia, treatment for patients with dementia places considerable financial and psychological pressure on families and caregivers (Chiao et al., 2015). Given the significant negative effects of dementia, it is critical to identify high-risk individuals at an early stage.

Some studies have adopted multiple perspectives to identify risk factors in people with MCI. A national cross-sectional study in China that comprised 46,011 older adults showed that MCI was associated with sociodemographic characteristics, including age, sex, parental history, education level, residence, and marital status (Jia et al., 2020a). Several cohort studies have shown a causal relationship between health status and behaviors that contribute to MCI. Chronic diseases, such as hypertension, stroke, and diabetes as well as harmful lifestyle behaviors, such as smoking and alcohol consumption, significantly increase the risk of MCI, while regular physical exercise, tea/coffee consumption, and playing Mahjong can prevent cognitive impairment (Kivipelto et al., 2018; Kakutani et al., 2019; Zhang et al., 2020, 2022). Owing to limitations in conventional regression methods in terms of collinearity potentially affecting predictors, some studies have applied machine learning based on imaging data or biomarkers to further determine whether an individual has MCI and to explore key features of MCI (Mirzaei et al., 2016; Wang et al., 2022; Alamro et al., 2023). However, most machine learning studies have only used single-wave panel data, and neurodegenerative disorders have a natural history of progression, thus ignoring the dynamic and longitudinal nature of these diseases, such that early identification and intervention could be sufficient.

Consequently, to address those deficiencies in previous studies, we used long short-term memory networks (LSTMs) in this study to capture the interdependence of predictors in longitudinal data. In combination with machine learning, it is possible to generate a model that can forecast the likelihood of conversion to MCI after several years. This model facilitates convenient and efficient screening for MCI and identification of risk groups for targeted intervention procedures. LSTMs are a form of recurrent neural network that address long-term dependencies and gaps between significant events in sequential data. Compared to traditional times series analysis like the Autoregressive Integrated Moving Average model (ARIMA), LSTMs models generally generate better outcomes in nonlinear and volatile time series data (Lou et al., 2022; Liu X. D. et al., 2023) despite the complexity of model interpretations and the long duration of model training. LSTMs were originally introduced into medically relevant applications to forecast the incidence and prevalence of diseases with considerable success during the COVID-19 pandemic (Borges and Nascimento, 2022; Gautam, 2022; Liu X. D. et al., 2023). Simultaneously, several studies have shown the feasibility of using LSTMs prediction in relation to individual characteristics in machine learning techniques to predict depression in older adults through applying longitudinal sequence data (Su et al., 2020; Lin et al., 2022).

No previous studies have used multiple sequence data waves to predict potential MCI in older Chinese adults. On the basis of the traits that LSTMs could effectively capture the temporal dependencies and trends of individual characters in longitudinal data from multiple data waves, and the capability that machine learning could extract important variables with significant trends related to MCI, therefore, this study assumes that the combination of LSTMs and machine learning could successfully identify the older adults at high risk for MCI and indicate instructions of implementing early interventions to prevent dementia.

## 2. Materials and methods

### 2.1. Data source and samples

The data used in this study were Waves 5–8 (2008, 2011, 2014, 2018) of the Chinese Longitudinal Healthy Longevity Survey (CLHLS), a secondary data series collected by the Center for Healthy Aging and Development and the China Mainland Information Group, Peking University, since 1998 (Center for Healthy Aging and Development Studies. The Chinese Longitudinal Healthy Longevity Survey (CLHLS)-Longitudinal Data, 1998–2018). Respondents in the CLHLS among the selected waves were randomly sampled from approximately half of the counties and city districts of China’s 23 mainland provinces. The CLHLS questionnaire includes a wide range of instruments, such as the Mini-Mental State Examination (MMSE), the Center for Epidemiologic Studies Depression Scale, and the Self-Rating Anxiety Scale. Previous studies have confirmed that the design of questionnaire and quality of datasets are excellent (Gu, 2008; Zeng, 2012).

The Wave 5 questionnaire of the CLHLS was used to obtain baseline characteristics of the older adults, including 2,334 home-based interviewees who continuously responded until Wave 8. After excluding respondents lacking answers or records for cognition measurement, that is, the MMSE questionnaire in this study, and respondents who had been diagnosed with dementia in Waves 5–7 based on the their MMSE scores, 2,128 eligible participants were included in the ultimate data preprocessing and statistical analysis.

### 2.2. Assessment of MCI and outcome variables

The MMSE has been widely applied to screen for cognitive dysfunction among older adults. In the CLHLS questionnaires, the MMSE was modified into a Chinese version, including 24 items within six dimensions: five items for orientation (five points in total), one for naming (seven points in total, one point for naming each kind of food), three for registration (three points in total), five for attention and calculation (five points in total), three for recall (three points in total), and seven for language (seven points in total). The final cognitive function score was the sum of the scores of the six dimensions, with a possible total of 30 points.

In this study, due to the age distribution of participants (age range, 70–80 years, 31.72%; age ≥ 80 years, 68.28%), MCI was defined as an MMSE score < 18 in this study (patients with MCI = 1; normal participants = 0) (An and Liu, 2016; Gao et al., 2017).

### 2.3. Predictors

We considered three levels of individual characteristics to fit the LSTMs and machine learning models from Waves 5–8, namely (Supplementary Table 1), (i) sociodemographic characteristics, such as age, sex, geographical area, education level, marital status, residence, income level, and living status; (ii) health behavior factors, including active smoking, alcohol consumption, exercise, self-rated health [SRH], and sleep quality; and (iii) health status factors, such as a history of hypertension, diabetes, cardiopathy, stroke, chronic respiratory disease, cancer, or gastrointestinal ulcer.

### 2.4. Processing of missing values

In order to reduce the probability of bias during the imputation procedure, variables with >20% information were abandoned to guarantee good performance (Jakobsen et al., 2017). The ultimate predictors included from CLHLS Waves 5–7 were imputed utilizing a MICE package in R studio 4.2.3 software, applying multivariate iterative random forest (“RF” method) imputation algorithms with five iterations to produce datasets with the least variance compared with datasets being imputed before.

### 2.5. Statistical analysis

Statistical analyses were performed using Keras package (version 2.6.0) software for deep learning and Scikit-Learn package (version 1.1.2) for machine learning in Python (version 3.9) software. We randomly partitioned the data into three disjoint sets: training, testing, and validation, with proportions of 60, 20, and 20%, respectively. Details about hyperparameters of LSTMs and parameters of three machine learning models were listed in Supplementary Tables 2, 3.

#### 2.5.1. The multivariate LSTMs models

**Figure 1:**
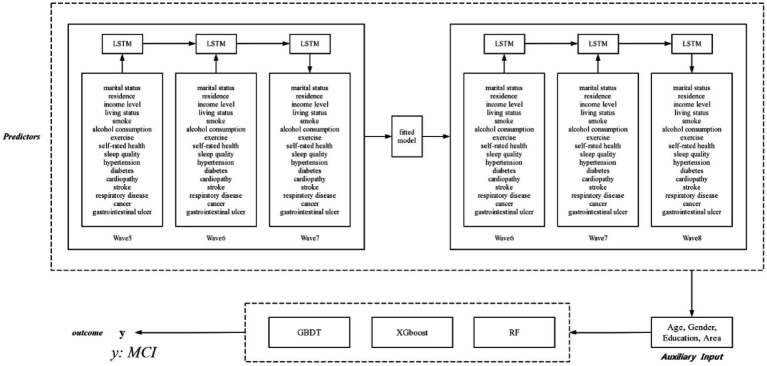
The predictors of the LSTMs model for older adults with MCI from CLHLS wave 5 to wave 8.

Machine learning techniques are generally applied to panel data from a cross-sectional perspective, but are not able to capture features with time sensitivity. To forecast the development of predictors and explore potential outcomes, recurrent neural networks (RNNs) are used to capture the inputs of predictors from specific time periods and transfer information to subsequent time periods through combining the interdependence among predictors. However, traditional RNNs cannot cope with gradient vanishing and gradient exploding in long-term dependency issues owing to their simple neuron structure, whereas LSTMs can successfully handle these disadvantages in RNNs through the use of “forget gate” and the sigmoid function in each LSTMs unit. The LSTMs model has been validated as a powerful and precise model for forecasting time-series data in longitudinal studies. As shown in Figure 1, time-sensitivity predictors in CLHLS Waves 5–6 were randomly split such that 70% of the samples were used to train the LSTMs model to forecast the values of the predictors in Wave 7, and the remaining 30% of the samples were used to test our LSTMs model. The model was then fitted to CLHLS Waves 6–7 to forecast predictors in Wave 8, combining invariable features such as age, sex, education level, and geographical area that did not need to be predicted over time to constitute a new dataset.

#### 2.5.2. Synthetic minority oversampling technique

Imbalanced data were a challenge for machine learning as the proportion of older adults with MCI was only 16.92% in this study. A common issue is that models tend to be biased toward the majority class, resulting in suboptimal performance. To address this problem, we applied the synthetic minority oversampling technique (SMOTE). SMOTE creates synthetic samples from the existing minority class through interpolation from its nearest neighbors, thereby increasing the number of minority samples in the datasets.

#### 2.5.3. Gradient boosting decision tree (GBDT)

The GBDT is an ensemble machine learning approach for classification and regression based on the CART algorithm. The GBDT improves prediction accuracy through gradually improving estimation using a boosting method. In addition, the GBDT utilizes a nonlinear regression procedure to improve tree accuracy. A series of decision trees was created, which produced a set of weak prediction models and generated loss functions. The final classification model was the weighted sum of all weak prediction models through each round of training.

#### 2.5.4. Extreme gradient boosting

XGBoost is a scalable and efficient implementation of gradient boosting, a popular machine learning technique that combines weak learners (typically decision trees) into a strong ensemble model. XGBoost offers several advantages over other gradient boosting frameworks, such as parallelization, regularization, and missing value handling. In addition, XGBoost can handle encoded categorical variables.

#### 2.5.5. Random Forest algorithm

Random Forest (RF) is a machine learning technique that builds an ensemble of decision trees and aggregates their predictions. RF can handle both classification and regression problems, as well as categorical and numerical features. It also provides measures of feature importance and variable selection. RF introduces randomness in two ways: by bootstrapping the training data for each tree, and by selecting a random subset of features for each split. To analyze the ultimate result, each decision tree was accessed in the final decision to obtain a reliable result. Based on majority selection for all decision trees, each sample was classified into two classes.

### 2.6. Model assessment

To assess the outcomes of each machine learning model, we calculated the area under the receiver operating characteristic curve (ROC; AUC) and sensitivity (equation 1), specificity (equation 2), accuracy (equation 3), and balanced accuracy (equation 4). True positives and true negatives indicate older adults who were correctly identified as patients with MCI or the normal cognitive function group, respectively; false positives and false negatives indicate older adults who were inaccurately identified as patients with MCI or the normal cognitive function group, respectively. Each machine learning model could predict the probability of cognitive impairment in older adults. If the probability of an individual was greater than the threshold, then older adults were regarded as patients with MCI, and vice versa. To further evaluate and understand the prediction models, we calculated the net benefit of the machine learning models using decision curve analysis (DCA). This method indicated the proportion of patients who received a correct diagnosis minus the percentage of patients who were misdiagnosed under different threshold values.


(1) $$\text{Sensitivity}=\frac{\text{True}\;\text{Positive}}{\text{True}\;\text{Positive}+\text{False}\;\text{Negative}}$$

(2) $$\text{Sensitivity}=\frac{\text{True}\;\text{Negative}}{\text{True}\;\text{Negative}+\text{False}\;\text{Positive}}$$

(3) $$\text{Accuracy}=\frac{\text{True}\;\text{Negative}+\text{True}\;\text{Positive}}{\begin{array}{l} \text{True}\;\text{Negative}+\text{True}\;\text{Positive}\\ +\text{False}\;\text{Negative}+\text{False}\;\text{Positive} \end{array}}$$

(4) $$\text{Balance accuracy}=\frac{2*\text{Specificity}*\text{Sensitivity}}{\text{Specificity}+\text{Sensitivity}}$$

### 2.7. SHapley Addictive explanation models

For ensemble machine learning models applied in this study, the processes of their predictions are generally opaque. Unlike the traditional statistical models, it is difficult for people to understand their working mechanisms and certain positive or negative contributions of predictors to the outcomes. To address this problem, post-hoc interpretations of the model output should be proposed for machine learning studies. Based on the individual and joint contributions among players, Shapley values are a way of fairly allocating the payoff of a game in cooperative game theory, which was introduced into machine learning techniques to explain the attribution of each input feature toward the outcome. SHapley Addictive explanation models (SHAP) is able to be used to provide various types of visualized explanations for machine learning models, including global feature importance, feature interaction, and feature dependence. SHAP was performed in Python using shap package (Version 0.42.1) in this study and was used to visualize the importance of each predictor and the association between predictors and MCI quantitatively (Ekanayake et al., 2022).

## 3. Results

As presented in Table 1, 2,146 older adults in the baseline CLHLS wave of 2008 participated in this study (older adults with MCI, 17.29%). The median age of patients with MCI was 92 years (range, 86–97 years), which was 10 years older than that of older adults with normal cognitive function (82 years, range, 78–88 years). The proportions of older adult males (46.62%) and females (53.38%) were relatively equal, with approximately two-thirds of the participants with MCI being female. Of older adults with MCI, 71.67% were illiterate, and 75.28% were single older adults. Older adults with low or very low-income levels comprised the majority of participants with MCI. The percentage of individuals living alone was higher among those with normal cognitive function than among those with MCI. Only 13.61% of older adults regularly exercised among those with MCI. People with normal cognitive function generally rated their health and sleep quality as better than those with MCI. A higher percentage of older adults in the normal group had a diagnosis of hypertension. A total of 14.17% of older adults with a history of stroke had poorer MMSE scores.

**Table 1:** Predicted characteristics in 2018 and odds ratio of older adults with MCI.

Predictors		All N (%)	MCI N (%)	Normal N (%)	Crude OR (95% CI)	Adjusted OR (95% CI)
Overall		2,128	360 (16.92)	1,768 (83.08)		
Sociodemographic variables						
Age		83 (78–90)	92 (86–97)	82 (78–88)	1.136 (1.119, 1.154)***	1.123 (1.103, 1.143)***
Gender	Male	992 (46.62)	117 (32.50)	875 (49.49)	Ref	Ref
	Female	1,136 (53.38)	243 (67.50)	893 (50.51)	2.035 (1.602, 2.586)***	1.331 (0.956, 1.854)
Geographical area	Eastern	959 (45.07)	155 (43.06)	804 (45.48)	Ref	Ref
	Central	471 (22.13)	82 (22.78)	389 (22.00)	1.093 (0.815, 1.467)	1.008 (0.712, 1.425)
	Northeastern	87 (4.09)	15 (4.17)	72 (4.07)	1.081 (0.604, 1.934)	1.749 (0.877, 3.489)
	Northwestern	611 (28.71)	108 (30.00)	503 (28.45)	1.114 (0.850, 1.459)	0.843 (0.611, 1.164)
Education	Literate	1,105 (51.93)	102 (28.33)	1,003 (56.73)	Ref	Ref
	Illiterate	1,023 (48.07)	258 (71.67)	765 (43.27)	3.316 (2.588, 4.249)***	1.641 (1.199, 2.247)**
Marital status	Married	933 (43.84)	89 (24.72)	844 (47.74)	Ref	Ref
	Single	1,195 (56.16)	271 (75.28)	924 (52.26)	2.781 (2.151, 3.596)***	1.292 (0.931, 1.794)
Residence	City	352 (16.54)	51 (14.17)	301 (17.02)	Ref	Ref
	Town/Rural	1776 (83.46)	309 (85.83)	1,467 (82.98)	1.243 (0.902, 1.714)	0.967 (0.651, 1.436)
Income level	Very high	52 (2.44)	3 (0.83)	49 (2.77)	Ref	Ref
	High	396 (18.61)	41 (11.39)	355 (20.08)	1.886 (0.563, 6.324)	2.306 (0.594, 8.949)
	Fair	1,467 (68.94)	267 (74.17)	1,200 (67.87)	3.634 (1.124, 11.747)*	3.426 (0.923, 12.725)
	Low	193 (9.07)	42 (11.67)	151 (8.54)	4.543 (1.348, 15.309)*	3.671 (0.939, 14.350)
	Very low	20 (0.94)	7 (1.94)	13 (0.74)	8.795 (1.993, 38.802)**	5.674 (1.067, 30.180)*
Living	With family	1732 (81.39)	309 (85.83)	1,423 (80.49)	Ref	Ref
	Alone	396 (18.61)	51 (14.17)	345 (19.51)	0.681 (0.495, 0.936)*	0.589 (0.405, 0.857)**
Health behavior/health status variables						
Smoking	Yes	345 (16.21)	36 (10.00)	309 (17.48)	Ref	Ref
	No	1783 (83.79)	324 (90.00)	1,459 (82.52)	1.906 (1.322, 2.747)***	1.086 (0.688, 1.712)
Alcohol consumption	Yes	327 (15.37)	32 (8.89)	295 (16.69)	Ref	Ref
	No	1801 (84.63)	328 (91.11)	1,473 (83.31)	2.053 (1.398, 3.014)***	1.390 (0.866, 2.231)
Exercising	Yes	711 (33.41)	49 (13.61)	662 (37.44)	Ref	Ref
	No	1,417 (66.59)	311 (86.39)	1,106 (62.56)	3.799 (2.769, 5.212)***	2.277 (1.596, 3.248)***
SRH	Very good	258 (12.12)	28 (7.78)	230 (13.01)	Ref	Ref
	Good	744 (34.96)	99 (27.50)	645 (36.48)	1.261 (0.807, 1.969)	1.104 (0.653, 1.868)
	Fair	824 (38.72)	155 (43.06)	669 (37.84)	1.903 (1.239, 2.924)**	1.562 (0.928, 2.631)
	Bad	262 (12.31)	64 (17.78)	198 (11.20)	2.655 (1.638, 4.304)***	2.069 (1.145, 3.740)*
	Very bad	40 (1.88)	14 (3.89)	26 (1.47)	4.423 (2.071, 9.448)***	3.874 (1.527, 9.826)**
Sleep quality	Very good	364 (17.11)	44 (12.22)	320 (18.10)	Ref	Ref
	Good	725 (34.07)	121 (33.61)	604 (34.16)	1.457 (1.006, 2.111)*	1.309 (0.847, 2.024)
	Fair	704 (33.08)	133 (36.94)	571 (32.30)	1.694 (1.173, 2.446)**	1.084 (0.698, 1.683)
	Poor	285 (13.39)	47 (13.06)	238 (13.46)	1.436 (0.921, 2.239)	1.012 (0.598, 1.714)
	Very poor	50 (2.35)	15 (4.17)	35 (1.98)	3.117 (1.576, 6.165)***	2.442 (1.083, 5.506)*
Hypertension	Yes	902 (42.39)	122 (33.89)	780 (44.12)	Ref	Ref
	No	1,226 (57.61)	238 (66.11)	988 (55.88)	1.540 (1.214, 1.953)***	1.278 (0.959, 1.703)
Diabetes	Yes	177 (8.32)	21 (5.83)	156 (8.82)	Ref	Ref
	No	1951 (91.68)	339 (94.17)	1,612 (91.18)	1.562 (0.976, 2.501)	1.091 (0.630, 1.890)
Cardiopathy	Yes	360 (16.92)	42 (11.67)	318 (17.99)	Ref	Ref
	No	1768 (83.08)	318 (88.33)	1,450 (82.01)	1.660 (1.177, 2.342)**	1.565 (1.035, 2.368)*
Stroke	Yes	253 (11.89)	51 (14.17)	202 (11.43)	Ref	Ref
	No	1875 (88.11)	309 (85.83)	1,566 (88.58)	0.782 (0.562, 1.088)	0.519 (0.347, 0.776)***
Respiratory disease	Yes	264 (12.41)	53 (14.72)	211 (11.93)	Ref	Ref
	No	1,864 (87.59)	307 (85.28)	1,557 (88.07)	0.785 (0.567, 1.087)	0.716 (0.487, 1.054)
Cancer	Yes	15 (0.71)	2 (0.56)	13 (0.74)	Ref	Ref
	No	2,113 (99.30)	358 (99.44)	1755 (99.27)	1.326 (0.298, 5.901)	0.673 (0.120, 3.783)
Gastrointestinal ulcer	Yes	85 (3.99)	9 (2.50)	76 (4.30)	Ref	Ref
	No	2043 (96.01)	351 (97.50)	1,692 (95.70)	1.752 (0.870, 3.529)	3.006 (1.314, 6.878)**

Bold font indicates statistical significance (*p < 0.05, **p < 0.01, ***p < 0.001).

For further descriptive analysis, odds ratios (ORs) for each predictor were evaluated using univariate and multivariate logistic regression analyses. Among sociodemographic variables, the analysis showed that age was a risk factor for MCI (adjusted OR [aOR] 1.123, 95% CI 1.103–1.143). Compared with literate older adults, illiterate older adults had a higher risk of developing MCI (aOR 1.641, 95% CI 1.199–2.247). Older adults with very low income levels had a higher risk of MCI than their wealthier counterparts (aOR 5.673, 95% CI 1.067–30.180). Among health behavior/health status variables, older adults who did not regularly exercise had a high risk of MCI (aOR 2.277, 95% CI 1.596–3.248). Older adults with poor or very poor self-rated health had a higher risk of MCI compared with those who had very good self-rated health (aOR 2.069, 95% CI 1.145–3.740 and aOR 3.874, 95% CI 1.527–9.826, respectively). Moreover, older adults with no history of stroke had a reduced risk of MCI (aOR 0.515, 95% CI 0.347–0.776).

**Figure 2:**
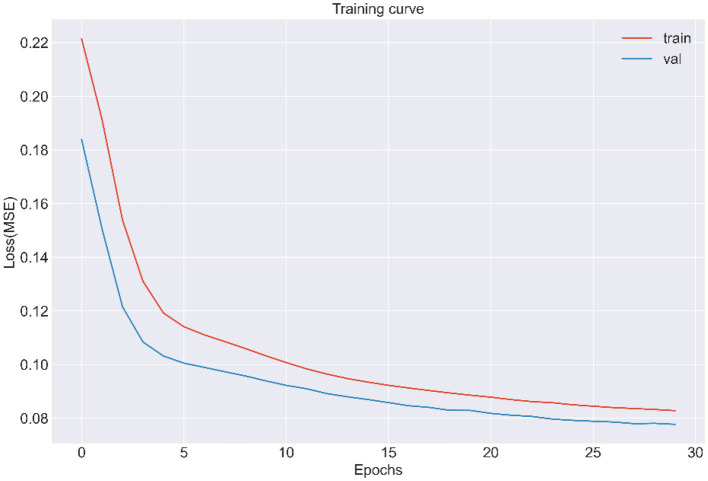
The training and validation curve of LSTMs from CLHLS wave5 to wave 7 (MSE, Mean squared error).

**Figure 3:**
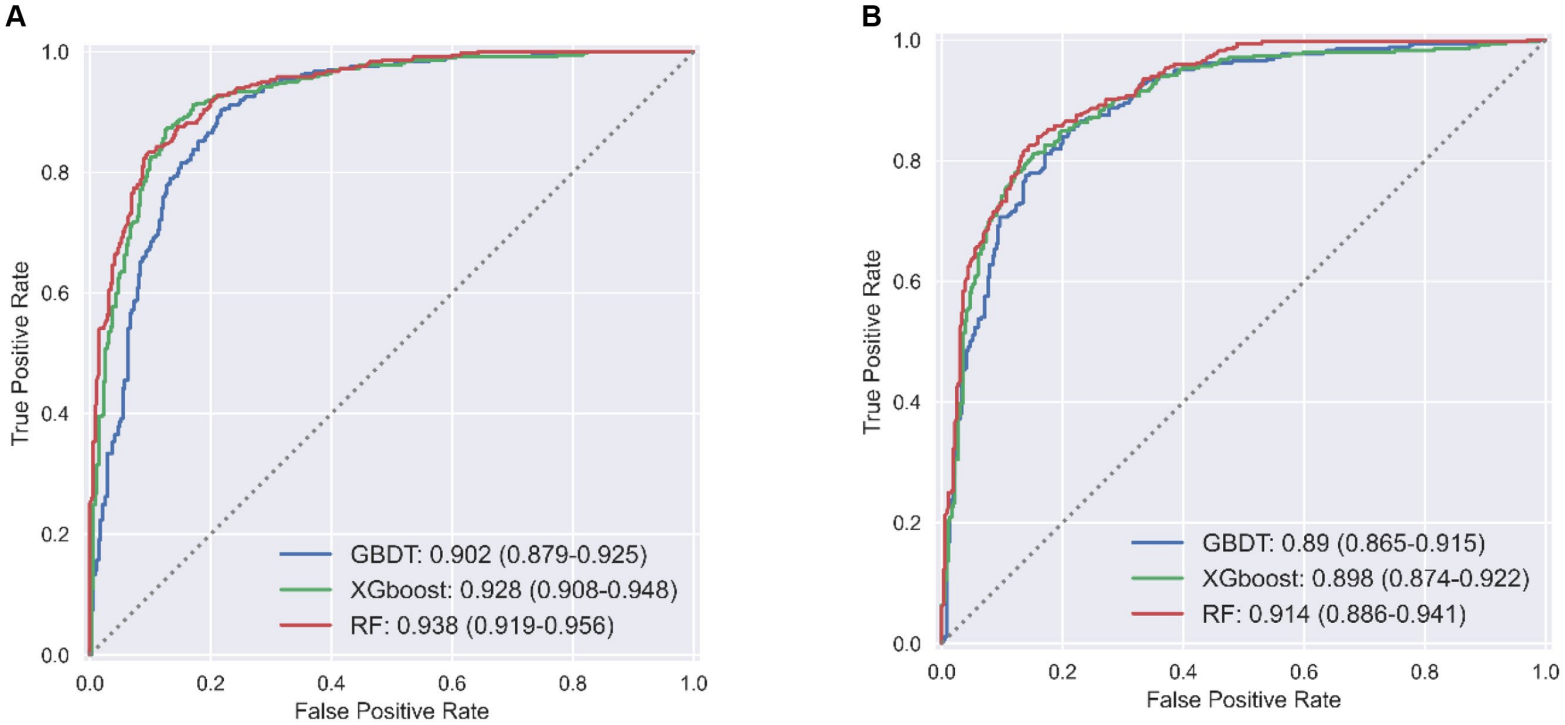
Performance of machine learning models in test set **(A)** and validation set **(B)**.

**Figure 4:**
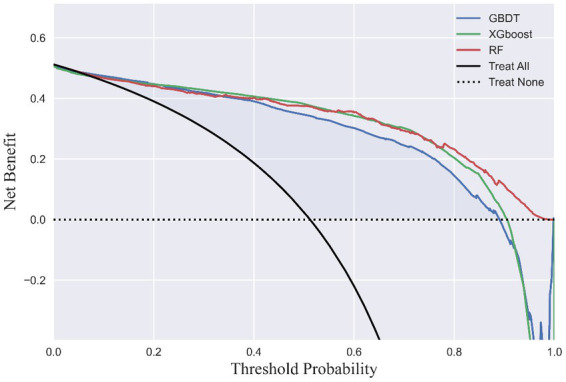
Decision curve analysis. The *x*-axis indicates the threshold probability of MCI. The *y*-axis indicates the net benefit.

LSTMs model performance is illustrated in Figure 2. The mean squared errors of both the training and validation sets were generally equal (approximately 0.08) after 30 rounds of training, and the inflection points of both sets were close, indicating that the LSTMs model could be utilized to forecast characteristics of older adults three years later. Table 2 and Figure 3A shows the ROC curves and AUC values of the three machine learning models in the testing set (GBDT 0.902, 95% CI 0.879–0.925; XGBoost 0.928, 95% CI 0.908–0.948; and RF 0.938, 95% CI 0.919–0.956). Table 3 and Figure 3B shows the performance of the three models in the validation set. The AUC values of all three machine learning models in the test sets were >0.9. The three machine learning models produced equal results in the validation sets, indicating that they were robust models for classifying patients with MCI and healthy people. XGBoost had the highest and most balanced accuracy and the second-highest sensitivity using 0.3 as a threshold (Table 2), and RF produced the highest sensitivity under this condition. The DCA results (Figure 4) showed that the XGboost and RF models were close, within the range of 0–0.8, and the net benefit values were higher than 0.4 using 0.3 as a threshold.

**Table 2:** Performance of machine learning models in test set of predicting MCI among Chinese older adults.

Model	AUC	Thresholds	TP/TN/FP/FN	Sensitivity (%)	Specificity (%)	Accuracy (%)	Balanced accuracy (%)
GBDT	0.902 (0.879, 0.925)	0.3	335/255/90/27	92.54 (89.84, 95.25)	73.91 (69.28, 78.55)	83.45 (80.71, 86.19)	85.13 (82.65, 87.62)
		0.4	325/270/75/37	89.78 (86.66, 92.90)	78.26 (73.91, 82.61)	84.16 (81.47, 86.85)	85.30 (82.79, 87.82)
		0.5	313/278/67/49	86.46 (82.94, 89.99)	80.58 (76.41, 84.75)	83.59 (80.86, 86.32)	84.37 (81.75, 86.98)
		0.6	303/286/59/59	83.70 (79.90, 87.51)	82.90 (78.93, 86.87)	83.31 (80.56, 86.06)	83.70 (81.01, 86.39)
		0.7	287/296/49/75	79.28 (75.11, 83.46)	85.80 (82.11, 89.48)	82.46 (79.66, 85.26)	82.23 (79.40, 85.07)
XGboost	0.928 (0.908, 0.948)	0.3	336/267/78/26	92.82 (90.16, 95.48)	77.39 (72.98, 81.81)	85.29 (82.68, 87.90)	86.60 (84.20, 88.99)
		0.4	332/277/68/30	91.71 (88.87, 94.55)	80.29 (76.09, 84.49)	86.14 (83.59, 88.69)	87.14 (84.76, 89.52)
		0.5	329/286/59/33	90.88 (87.92, 93.85)	82.90 (78.93, 86.87)	86.99 (84.51, 89.47)	87.73 (85.39, 90.08)
		0.6	321/292/53/41	88.67 (85.41, 91.94)	84.64 (80.83, 88.44)	86.70 (84.20, 89.21)	87.23 (84.82, 89.64)
		0.7	315/301/44/47	87.02 (83.55, 90.48)	87.25 (83.73, 90.77)	87.13 (84.66, 89.60)	87.38 (84.95, 89.80)
RF	0.938 (0.919, 0.956)	0.3	349/212/133/13	96.41 (94.49, 98.33)	61.45 (56.31, 66.59)	79.35 (76.37, 82.33)	82.70 (80.15, 85.25)
		0.4	340/260/85/22	93.92 (91.46, 96.38)	75.36 (70.82, 79.91)	84.87 (82.22, 87.51)	86.40 (84.01, 88.80)
		0.5	317/292/53/45	87.57 (84.17, 90.97)	84.64 (80.83, 88.44)	86.14 (83.59, 88.69)	86.61 (84.15, 89.08)
		0.6	300/313/32/62	82.87 (78.99, 86.75)	90.72 (87.66, 93.79)	86.70 (84.20, 89.21)	86.46 (83.91, 89.00)
		0.7	247/327/18/115	68.23 (63.44, 73.03)	94.78 (92.44, 97.13)	81.19 (78.31, 84.07)	78.79 (75.59, 81.99)

GBDT, Gradient boosting decision tree; XGboost, Extreme gradient boosting; RF, Random Forest; AUC, Area Under the Curve; TP, True Positives; TN, True Negatives; FP, False Positives; FN, False Negatives.

**Table 3:** Performance of machine learning models in validation set of predicting MCI among Chinese older adults.

Model	AUC	TP/TN/FP/FN	Sensitivity (%)	Specificity (%)	Accuracy (%)	Balanced accuracy (%)
GBDT	0.890 (0.865, 0.915)	304/266/98/40	88.37 (84.98, 91.76)	73.08 (68.52, 77.63)	80.51 (77.71, 83.54)	81.50 (78.72, 84.29)
		300/273/91/44	87.21 (83.68, 90.74)	75.00 (70.55, 79.45)	80.93 (78.16, 83.94)	81.63 (78.83, 84.43)
		297/282/82/47	86.34 (82.71, 89.97)	77.47 (73.18, 81.76)	81.78 (79.06, 84.73)	82.16 (79.37, 84.95)
		292/289/75/52	84.88 (81.10, 88.67)	79.40 (75.24, 83.55)	82.06 (79.36, 85.00)	82.14 (79.32, 84.95)
		284/302/62/60	82.56 (78.55, 86.57)	82.97 (79.11, 86.83)	82.77 (80.11, 85.66)	82.32 (79.47, 85.17)
XGboost	0.898 (0.874, 0.922)	309/251/113/35	89.83 (86.63, 93.02)	68.96 (64.20, 73.71)	79.10 (76.10, 82.09)	80.68 (77.88, 83.47)
		301/269/95/43	87.50 (84.01, 90.99)	73.90 (69.39, 78.41)	80.51 (77.71, 83.54)	81.35 (78.55, 84.16)
		295/282/82/49	85.76 (82.06, 89.45)	77.47 (73.18, 81.76)	81.50 (78.76, 84.47)	81.83 (79.02, 84.65)
		282/295/69/62	81.98 (77.91, 86.04)	81.04 (77.02, 85.07)	81.50 (78.76, 84.47)	81.15 (78.24, 84.06)
		271/302/62/73	78.78 (74.46, 83.10)	82.97 (79.11, 86.83)	80.93 (78.16, 83.94)	80.06 (77.05, 83.07)
RF	0.914 (0.886, 0.941)	329/226/138/15	95.64 (93.48, 97.80)	62.09 (57.10, 67.07)	78.39 (75.36, 81.42)	81.13 (78.44, 83.83)
		310/261/103/34	90.12 (86.96, 93.27)	71.70 (67.08, 76.33)	80.65 (77.74, 83.56)	81.90 (79.16, 84.64)
		290/303/61/54	84.30 (80.46, 88.15)	83.24 (79.40, 87.08)	83.76 (81.04, 86.47)	83.45 (80.69, 86.22)
		269/318/46/75	78.20 (73.83, 82.56)	87.36 (83.95, 90.78)	82.91 (80.14, 85.68)	81.64 (78.68, 84.59)
		233/339/25/111	67.73 (62.79, 72.67)	93.13 (90.53, 95.73)	80.79 (77.89, 83.69)	77.41 (74.07, 80.75)

GBDT, Gradient boosting decision tree; XGboost, Extreme gradient boosting; RF, Random Forest; AUC, Area Under the Curve; TP, True Positives; TN, True Negatives; FP, False Positives; FN, False Negatives.

**Figure 5:**
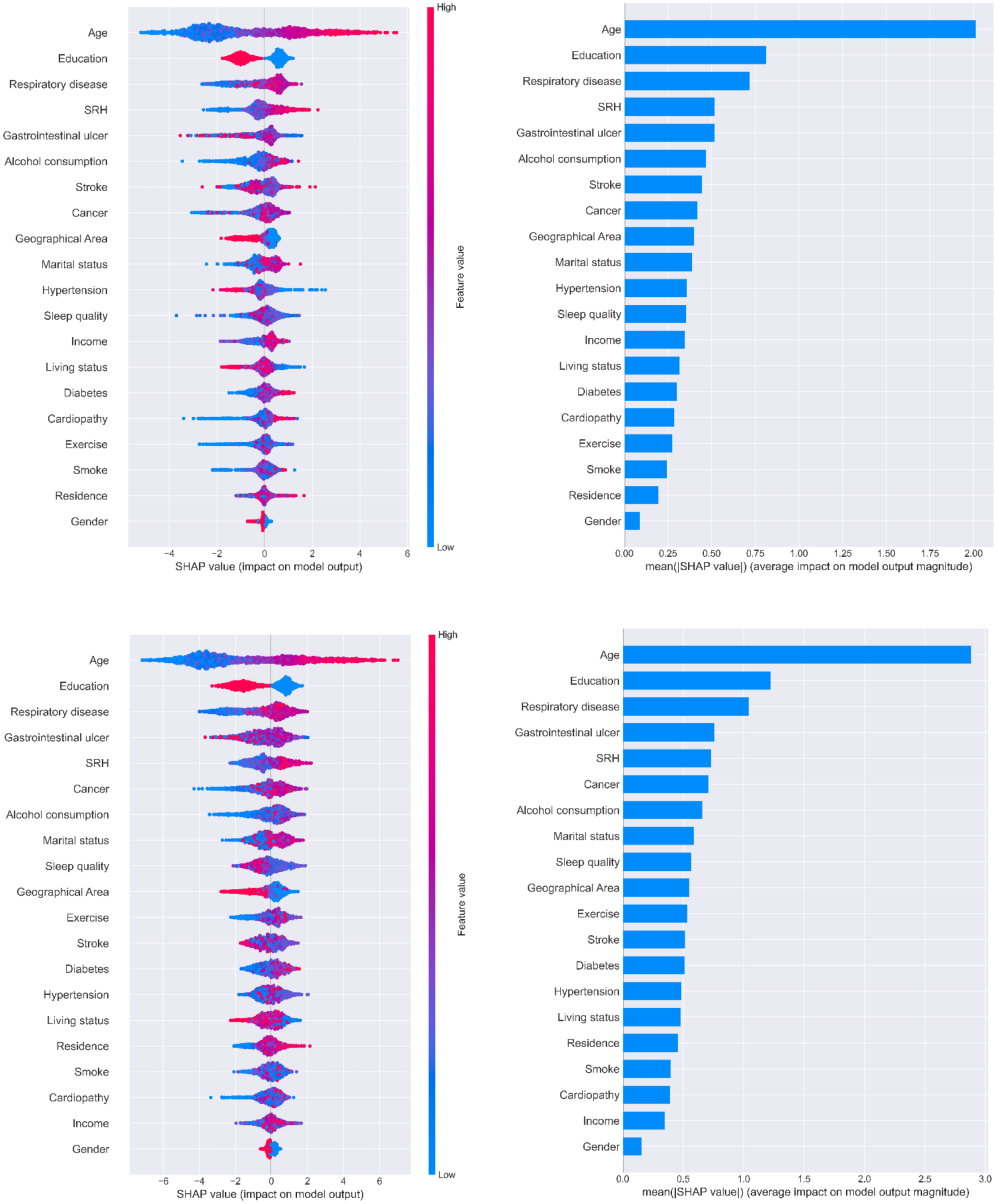

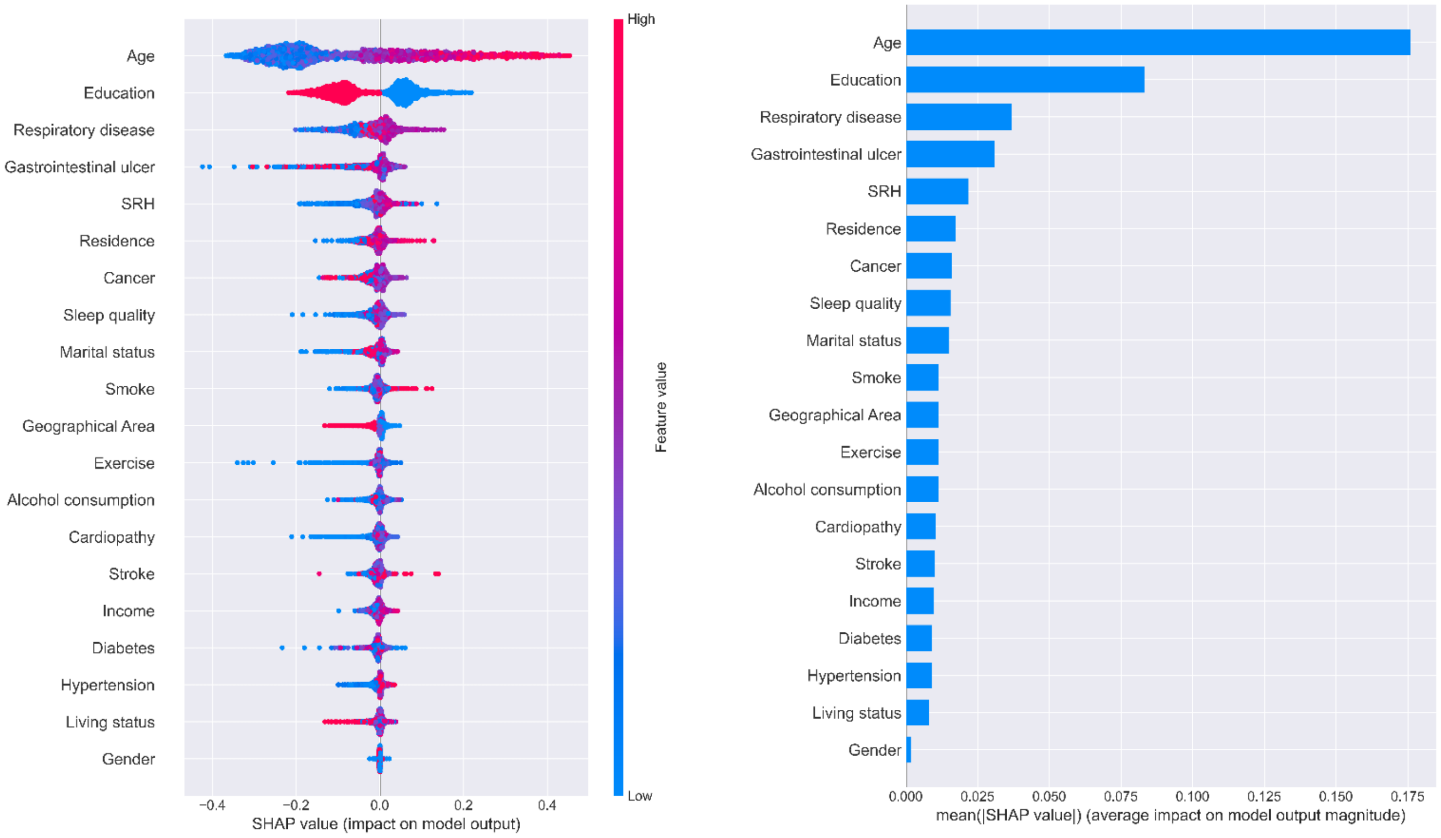
Importance of predictors analysis by SHAP model. SHAP (SHapley Additive exPlanation) values are ranked by value of a feature to the predictions made by the GBDT/XGboost/RF.

Figure 5 illustrates the ranking of feature importance in MCI prediction. Age, education, and chronic respiratory disease were the first, second, and third-most important characteristics of older adults when predicting MCI in all three models, respectively. Younger literate older adults with no history of chronic respiratory disease had a lower probability of developing MCI. Self-rated health was also an important feature that presented a direct trend in MCI output. All three SHAP models indicated that having a gastrointestinal ulcer was one of the most important features for predicting potential MCI in patients; however, it did not show a clear tendency in relation to MCI progression.

## 4. Discussion

**Figure 6:**
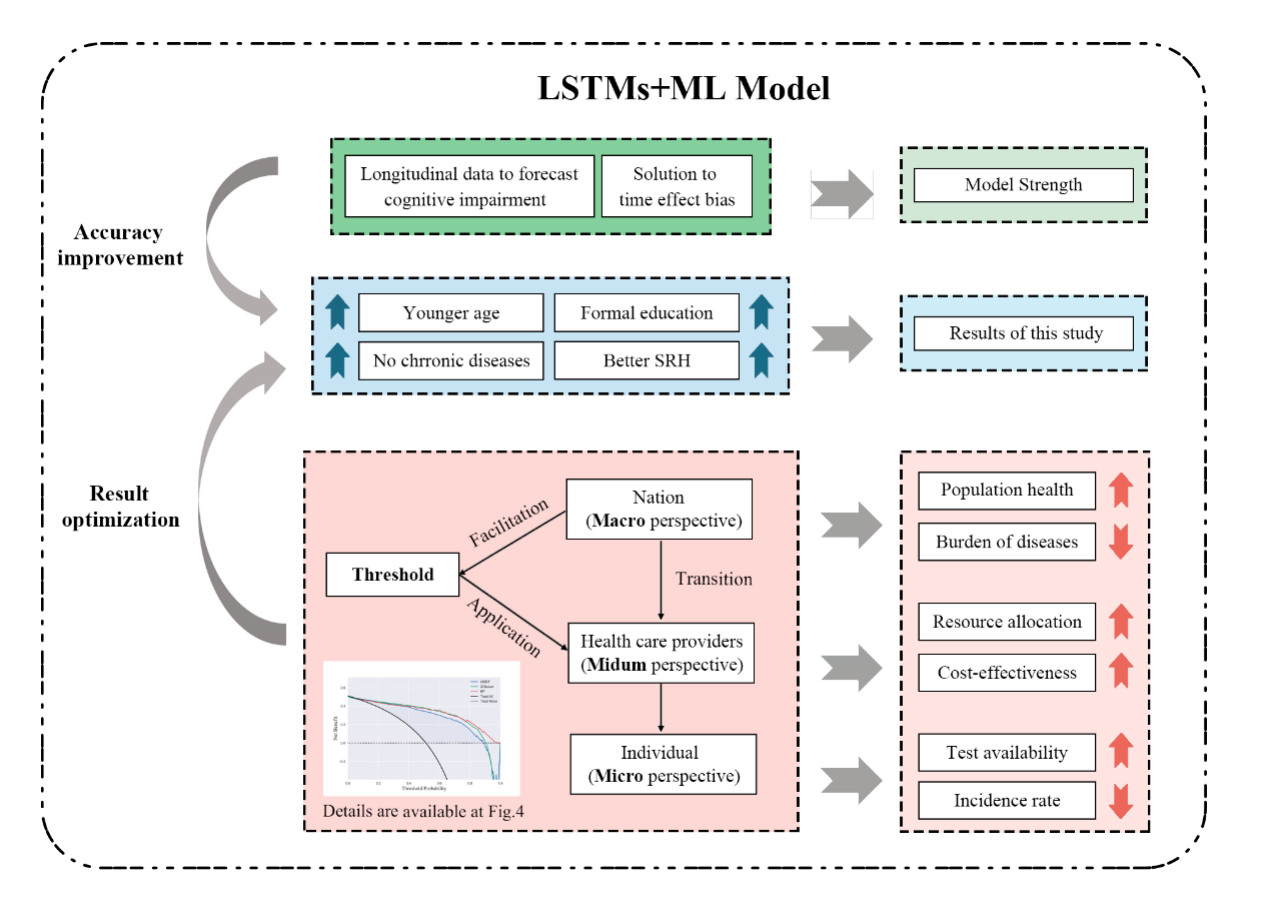
Conceptual framework of discussion in this study.

To our knowledge, this study is the first to forecast cognitive impairment in older Chinese adults using an LSTMs model and machine learning based on CLHLS Waves 5–8, with predictions that included sociodemographic health behaviors and health status characteristics. In total, 2,128 older adults were included in this study. Our LSTMs model produced robust results in the validation set; thus, it was capable of forecasting the feature values of older adults in the next wave using the SMOTE algorithm and three machine learning approaches that performed well in predicting MCI. Figure 6 depicts the conceptual framework discussed, summarizes the accuracy of the prediction models, presents the results, and presents multiple perspective values.

Regarding model precision, this prediction method combining LSTMs and machine learning can be successfully applied to longitudinal data to capture temporal information, thus improving the accuracy of MCI predictions in older adults (Chae et al., 2018; Wang et al., 2019; Su et al., 2020). To date, most studies have used LSTMs to forecast the prevalence and incidence rates or temporal trends in medical-related applications (Borges and Nascimento, 2022; Gautam, 2022; Liu X. D. et al., 2023). In addition, LSTMs have shown excellent performance when predicting high-dimensional data such as air and water pollution (Kim et al., 2022; Middya and Roy, 2022). Thus, building on previous LSTMs applications, some studies have used LSTMs to detect early health deterioration in individual clinical data (da Silva et al., 2021). Furthermore, the utilization of LSTMs to forecast individual features, followed by machine learning to construct predictive models, has been shown to be useful in disease prediction; for example, in the prediction of depression (Su et al., 2020; Lin et al., 2022) and in glaucoma assessment (Dixit et al., 2021). To date, no studies have utilized LSTMs and machine learning to establish a prediction model for MCI and explore its risk factors. Compared to the previous two prediction models using CLHLS, this study revealed relatively high accuracy and robustness with the AUCs of 0.902 to 0.938 for the test set and high sensitivity and specificity, and from 0.890 to 0.914 for the validation test. One longitudinal study proposed to use The Growth Mixed Model (GMM) and machine learning combination to forecast the MMSE trajectory of older adults. Due to the time effect bias for the application of constant baseline individual character in forecasting models, the AUCs of their models ranged from 0.51 to 0.66 in eight machine learning techniques (Wu et al., 2022). The other study utilized sociodemographic and life behavioral features of Chinese older adults to construct prediction models, achieving an accuracy of 0.7540 and the AUC of 0.8269 at maximum (Wang et al., 2022). To conclude, the outcomes of LSTMs and machine learning framework demonstrates the feasibility and effectiveness of the study hypothesis.

Three decision tree-based models (GBDT, XGBoost, and RF) were used with SHAP to interpret individual predictions. Age, education level, chronic respiratory disease, gastrointestinal ulcers, and self-rated health were identified as the five most important predictors in this study. Age and education level have been reported in previous studies to be important predictors of MCI (Chun et al., 2022; Liu H. et al., 2023). Physiological decline in cognitive function is inevitable as people age (Langa and Levine, 2014) and age is a major predictor of MCI. Lower educational levels have been shown to be significantly associated with cognitive decline, and education in later life may also contribute to improved cognitive function (Peeters et al., 2020). According to our results, older adults with a formal education performed well in terms of MMSE scores. The other three features were not found to be strong predictors in other studies; however, they have all been shown to be closely associated with MCI. Older adults with no history of chronic respiratory disease are less likely to develop MCI. Common chronic respiratory diseases, such as chronic obstructive pulmonary disease and obstructive sleep apnea-hypopnea syndrome, lead to perennial hypoxia and hypercarbia (Olaithe et al., 2018), causing damage to brain functions, including language, execution, and attention. Ultimately, cognitive function continues to decline under these pathological conditions. Gastrointestinal ulcers did not show a clear trend in Figure 5, whereas changes in metabolic substances in the gastrointestinal tract under pathological conditions are reported to impair brain function via the gut-brain axis (Zeng et al., 2022). Moreover, a healthy gastrointestinal tract can guard against cognitive decline and mitigate neuroinflammation (Xiang et al., 2022); hence, this result needs to be verified in another study. The SHAP analysis illustrated a positive correlation between self-rated health and MCI; that is, good self-rated health may represent good cognitive function and vice versa, which is consistent with previous cohort studies (Bond et al., 2006).

MCI prediction models could provide references for clinical practice and bring broad benefits to society; however, they still need adjustment and practice to meet the standards for real-world application. When applied for MCI screening, the most appropriate prediction model requires striking a balance between sensitivity and specificity to achieve high precision and cost-effectiveness. Consequently, it is critical to determine the threshold for identifying patients with MCI and conducting further interventions. As shown in Figure 4, the XGBoost prediction model had the greatest net benefit and balanced accuracy when the threshold probability was <0.6. When the threshold probability was 0.3, RF had the highest sensitivity and identifies most patients with MCI with relatively low cost-effectiveness owing to the proportion of misdiagnoses. Determining the ultimate thresholds require constant evaluation and collaboration between governments and healthcare providers to obtain optimal clinical, economic, and social outcomes.

Ongoing application of this approach and cooperation can be viewed from three perspectives: the nation (macro), healthcare providers (medium), and individuals (micro). As a macro-regulator, the government should enhance the utilization of big data and incorporate prediction models into various healthcare provider and public Internet platforms. This screening method could promote population health and reduce the disease burden. Various healthcare providers can select different thresholds in terms of specific medical conditions and testing technologies and change their criteria according to local prevalence and incidence. As psychiatric hospitals are generally equipped with adequate medical resources, the threshold for machine learning models could be relatively low to achieve suitable resource allocation. Once MCI predictive models become more sophisticated with continuous training and with more individual information available, such as risk genes or biomarkers, the threshold can be adjusted to pursue relatively high cost-effectiveness. In terms of the micro perspective, the general public could benefit through becoming more aware of their own and their families’ risk of MCI through the application of this prediction model, avoiding additional examinations and ameliorating individual MCI risk.

This study contributes to the prevention of MCI and dementia. First, the combination of an LSTMs model and machine learning could precisely identify patients with MCI and their critical features several years earlier. Age, literacy level, chronic respiratory disease, gastrointestinal ulcers, and self-rated health were good predictors of MCI. Second, MCI prediction models have substantial clinical, economic, and social value through optimizing prediction under governmental direction and adjusting thresholds for MCI probability according to the specific needs of different healthcare providers. Finally, this study contributes to the prevention of dementia and MCI and promotes healthy aging.

## 5. Study limitations

This study had some limitations. First, we examined the robustness of both LSTMs and machine learning models and included four waves of data; however, our findings need to be validated in another cohort. Lacking external validation may affect the performance and adaptability of prediction models in different scenarios, as well as the confidence in the predictive ability of the models. Therefore, future researchers need to use other sources or types of data to validate this method framework and explore possibilities for improvement. Second, most predictors in this study were self-reported, which could have led to information bias. Third, the MMSE has a ceiling effect, meaning that it may not detect subtle changes in cognition that occur during MCI. Furthermore, MMSE scores could be affected by certain individual sociodemographic background factors (Arevalo-Rodriguez et al., 2021; Wu et al., 2022); therefore, MCI evaluations should be more comprehensive and include using Montreal Cognitive Assessment and the Clinical Dementia Rating evaluations, in addition to detecting biomarkers and undertaking imaging examinations for a more accurate clinical diagnosis in future studies. While this study proposes a convenient screening method using accessible individual features for the general public, outcomes obtained using this method are for reference only and cannot replace acknowledged MCI diagnosis standards.

## 6. Conclusion

This study showed that individual features could be predicted through combining LSTMs and machine learning models. The risk of MCI could be accurately predicted through exploring critical risk factors, such as age, education level, chronic respiratory disease, gastrointestinal ulcer, and self-rated health, in patients with MCI using three SHAP models among older Chinese adults based on four waves of CLHLS datasets. The combination of LSTMs and machine learning models captured the interdependence of predictors and generated an effective decision support system for healthcare providers to identify patients at high risk of MCI. With macro-direction undertaken at a governmental level, this screening method can continue to be optimized to obtain better thresholds for MCI screening. Our study findings may offer healthcare providers MCI screening support to implement early interventions to delay the progression from MCI to dementia, increase test availability among the population, and reduce incidence rates and treatment costs, ultimately contributing to healthy aging.

## Data availability statement

The original contributions presented in the study are included in the article/Supplementary material, further inquiries can be directed to the corresponding authors.

## Author contributions

YH: Conceptualization, Writing – original draft, Writing – review & editing, Data curation, Formal analysis, Investigation, Methodology, Project administration, Software, Validation, Visualization. ZH: Conceptualization, Data curation, Formal analysis, Investigation, Project administration, Validation, Visualization, Writing – original draft, Writing – review & editing. QY: Validation, Writing – review & editing, Methodology, Software. HJ: Methodology, Software, Writing – review & editing, Data curation. TX: Writing – review & editing. YF: Writing – review & editing, Visualization. YZ: Writing – review & editing, Formal analysis. XZ: Writing – review & editing, Funding acquisition, Resources. CC: Funding acquisition, Resources, Writing – review & editing, Conceptualization, Investigation, Project administration, Supervision, Writing – original draft.
