# Supplementary material for: Predicting mild cognitive impairment among Chinese older adults: a longitudinal study based on long short-term memory networks and machine learning
Source: Front Aging Neurosci. 2023 Oct 23;15:1283243. doi: 10.3389/fnagi.2023.1283243 (PMC10626462; doi:10.3389/fnagi.2023.1283243)
Supplement: Supplementary file 1 [file Data_Sheet_1.docx]

**Supplementary Table 1. Details of predictor used in this study**

| **Variables** | **Category** |
| --- | --- |
| Age, years | ---- (Continuous Variable) |
| Gender | Male; Female |
| Geographical Area | **Eastern** (Beijing Tianjin Hebei Shanghai Jiangsu Zhejiang Fujian Shandong Guangdong Hainan)  **Central** (Shanxi Anhui Jiangxi Henan Hubei Hunan) |
|  | **Northeastern** (Liaoning Jilin Heilongjiang)  **Northwestern** (Guangxi Chongqing Sichuan Shanxi) |
| Education | Illiterate - 0 year of schooling;  Literate – schooling for at least 1 year |
| Marital status | Single (never married/divorced/separated and widowed); Married (married/married but not live together) |
| Residence | City; Town/Rural |
| Income level | Very high: 100000+; High: 75000 to 100000;  Fair: 50000 to 75000; Low: 25000 to 50000;  Very low: Less than 25000 |
| Living | With family; Alone |
| Smoking | Never (No); Ever (Yes) |
| Alcohol consumption | Never (No); Ever (Yes) |
| SRH (Self-rated health) | Very good; Good; Fair; Poor; Very Poor |
| Sleep quality | Very good; Good; Fair; Poor; Very Poor |
| Hypertension | Yes; No |
| Diabetes | Yes; No |
| Cardiopathy | Yes; No |
| Stroke | Yes; No |
| Respiratory disease | Yes; No |
| Cancer | Yes; No |
| Gastrointestinal ulcer | Yes; No |

**Supplementary Table 2. Hyperparameters of the LSTMs model in this study**

| **Hyperparameter** | **Value** | **Remark** |
| --- | --- | --- |
| Learning rate | 0.01 | Iteration steps |
| Batch size | 128 | Gradient descent method update weight w and deviation b |
| Activation function | “relu | f(x) = max (0,x) |
| Epoch | 30 | The total number of iterations is 2128*0.7/128*30 |
| LSTMs unit numbers | 30 | 30 data |
| LSTMs unit dimensions | 15 | Independent variable dimension |
| Loss | “categorical_crossentropy” | $L(y,\hat{y})=-sum_{i=1}^{n}y_{i}log(\hat{y_{i}})$ |

| Supplemental Table 3. Parameters of three machine learning models | |
| --- | --- |
| **Parameters** | **Value** |
| **GBDT** | |
| objective | “binary:logistic” |
| max_depth | 3 |
| max_features | Auto |
| min_samples_leaf | 1 |
| min_samples_split | 2 |
| n_estimators | 1000 |
| **XGBoost** | |
| objective | “binary:logistic” |
| max_depth | 6 |
| max_features | Auto |
| min_samples_leaf | 1 |
| min_samples_split | 2 |
| n_estimators | 1000 |
| **Random Forest** | |
| criterion | Gini index |
| max_depth | 6 |
| max_features | Auto |
| min_samples_leaf | 1 |
| min_samples_split | 2 |
| n_estimators | 1000 |
| bootstrap | True |
